# Physical education curriculum systematizing based on action research: a collaborative network between teacher-researchers from public schools in Quixadá — Ceará, Brazil

**DOI:** 10.3389/fspor.2025.1373271

**Published:** 2025-01-31

**Authors:** Rener Victor Oliveira de Souza, Maurício Teodoro de Souza, Luciano Nascimento Corsino, Willian Lazaretti da Conceição, Carla Ulasowicz, Luciana Venâncio, Luiz Sanches Neto

**Affiliations:** ^1^ Postgraduate Program in Physical Education, Federal University of Rio Grande do Norte (PPGEF-UFRN), Natal, Brazil; ^2^ Professional Postgraduate Program in Physical Education in National Network, Federal University of Ceará (ProEF-UFC), Fortaleza, Brazil

**Keywords:** school physical education, pedagogical practice, complexity, convergences, curriculum, high school, collaboration

## Abstract

For more than forty years, physical education has been associated with human sciences in Brazil, with an emphasis on the dynamics of culture. This renewing movement challenged the hegemony of sport and health as predominant themes in the school physical education curriculum, as well as pointing out dilemmas to the positivist scientific paradigm. In this sense, advances in theoretical-methodological propositions in Brazilian physical education are intertwined with scientific understandings in the area. Twenty years ago, from a complex epistemological perspective, a curricular systematization—proposed by an autonomous community of teachers-researchers—brought these understandings closer together, valuing the convergence between the dynamics of culture, movement, the body and the environment. In this research, our objective is to analyze how this systematization can contribute to the pedagogical practice of physical education teachers in the public high school network in the city of Quixadá, located in the interior of the state of Ceará, Brazil. Our qualitative itinerary has action research as a method of intervention, including class observations, group meetings and field diary. Data has been analyzed through thematic analysis, characterizing a collaborative network between participants. We identified that curricular documents direct subject content towards the predominance of cultural elements. This direction, in turn, can make it difficult to develop intersubjective knowledge related to the body, movement and the environment. However, when dialoguing with the systematization and reflecting on the curriculum, the thematic subject content blocks make it possible to visualize (inter)personal aspects, movements and environmental demands as complex knowledge specific to physical education.

## Introduction

We understand physical education as a curricular component of schooling in a broad and complex sense, valuing its historical process and problematizing the various power struggles that permeate it, encompassing policies and knowledge. Among multiple influences, we initially highlight the elaboration of concepts about the body and its use as a labor force mediated by capitalism, with remnants of Black slavery (1), as well as the emphasis on physical exercises to produce agile bodies and strong under the logic of hygiene and militarism (2). This problem was limited to physical education in the first half of the 20th century and culminated in the hegemonic shift from gymnastics to sport as the predominant subject content in its second half.

Another important milestone is the development of new pedagogical concepts mobilized by the democratic movement in Brazil, breaking the binomial of sport and physical fitness related to biological health as the main scientific contribution in the area. This renewing context is marked mainly by theoretical-methodological propositions from the end of the 1970s, when postgraduate studies in physical education began in the country (3). Taking the empirical basis of this process as an indication of the specificity of physical education—and his pedagogical practice as a teacher-researcher of physical education in basic education –, Sanches Neto (4) systematized a curricular proposal based on the complexity, convergence and integration of four entangled dynamics: culture, movement, body and environment. As part of the curricular principles, the thematic subject content blocks—cultural elements, movements, (inter)personal aspects and environmental demands—were deepened by an autonomous community of teacher-researchers (5–7).

In addition to the autonomous community that involved members from the five Brazilian regions, this systematization perspective was critically analyzed by other teacher-researchers (8). For example, Betti (9, 10) and Freire (11)—in the Brazilian context—and Ovens and Butler (12)—in the international context—pointed out advances in this theoretical-methodological proposal based on complexity. However, these advances do not necessarily go hand in hand with facing the challenges that each school faces on a daily basis, such as the emphasis on sport within the school physical education curriculum (13), or the multiple reflections on how to intervene in pedagogical practice (14). There are different modes of intervention carried out by physical education teachers, making it difficult to assign criteria to assess their coherence.

On the one hand, the overvaluation of bodily practices in physical education curricular guidelines—such as the National Common Curricular Base (BNCC) (15)—devalues students' learning and the complexity of their knowledge, as they are not consistent with their conditions of origin and life stories (16). It is necessary to value living circumstances and experiences, so practice is not enough to achieve physical education critical goals because practicing is not synonymous with living or experimenting. On the other hand, we assume that there are distances between school and university, researcher and teacher, configuring two parallel universes (17). Communication is essential to break these dualisms. As Freire (18) states, teaching requires research and there is no teaching without research and no research without teaching. The proposed systematization of teacher-researchers allows for common language practices in teaching and learning physical education (19).

The realities of Brazilian teachers face adversities and work overloads, influencing their absence, in addition to the lack of incentive for their continued education (20). The complexity of systematization is anchored in ongoing (self)educative and collaborative processes as presuppositions for the work of physical education teachers, through criticism of continuing education policies as a privilege for few teachers. The question that guides us is: How can curricular systematization—based on the convergence of knowledge—contribute to the pedagogical practice of physical education teachers in Quixadá? From this perspective, our general objective was to analyze how the systematization of thematic content blocks can contribute to the pedagogical practice of high school physical education teachers from the public basic education network in the municipality of Quixadá, state of Ceará, Brazil. The specific objectives were to (1) verify the complexity thinking in the pedagogical practice of high school teachers; (2) identify both curricular documents and knowledge that guide the teachers' pedagogical practice; and (3) present the proposal for systematizing thematic content blocks, with a view to understanding how the theoretical perspective can be constituted in the teachers' classes. Next, we address how complexity thinking is conceptualized, and what its relationship is with the systematization of thematic content blocks for school physical education.

## Complexity thinking and school physical education

This research—originally derived from a Master's thesis (21)—was guided by a perspective of complexity (22) in education and of complexity thinking in school physical education (7, 23). Being a teacher is full of characteristics that involve complexity in teaching practice, but then, what would complexity be and what is its relationship with school physical education? Morin (22) explains that complexity is a network of interactions with traces of disorder and uncertainty, a non-linear relationship different from Cartesian logic, which implies the process of self-organization of open systems. This process requires weaving together without the establishment of hierarchies, which could make peripheral relationships invisible in the organization of the system (22).

According to Ovens and Butler (12), complexity thinking has been the predominant line of theorization in physical education, assuming reality as chaotic and unpredictable. Sanches Neto et al. (7, p. 3) rely on “four concepts of complexity thinking” to explain how teacher-researchers collaborate autonomously within a knowledge community. (1) Relational connections, which allow sensitivity to the dynamics of social collectives and their changes as they move through space and time. (2) The interaction of teacher-researchers due to a common connection of interests, such as beliefs and practices that underlie decision-making and drive affective forces. (3) The influence of participation in a social collective, which provides opportunities for new actions in the sociocultural environment. (4) Proactivity as a relational quality aimed at the ability to act in the system that the teacher-researcher is part of.

From an educational point of view, Ovens et al. (23) state that complexity thinking emerged as responses to theoretical perspectives that limited the understanding of educational phenomena. There is an epistemological problem because complexity cannot be ruled out. However, there is a “modern pathology” (22, p. 15), which is the need to simplify or decomplexify a phenomenon. Betti (9, 10), when talking about the BNCC, criticizes the attempt to make the perspectives of physical education more “lean” in the school curriculum. In this sense, both Morin (22) and Betti (9, 10) denounce hyper-simplification. Venâncio and Sanches Neto (24) also warn about the simplification of school physical education and seek complexity to support pedagogical practice. Therefore, it is necessary to avoid intellectual simplism to develop a critical and reflective practice, with emphasis on subjects that have historically been intentionally silenced, such as the Black population's and Indigenous peoples' issues (24).

Complexity is reflected in teachers' pedagogical policies and practices, even if they do not identify their characteristics (23). As teacher-researchers, we need to problematize teaching, transgress the curriculum and value historically developed knowledge, especially that which has been made invisible (24). Looking at how these aspects are related to students' learning allows us to understand the teachers' work in school physical education. Students promote dynamic changes as they both individually and collectively affect a certain group or class. They interact through affectivity, share sociocultural connections and make reflections that generate actions, which is similar to the professional learning of teachers in knowledge communities (7, 19). Then, complexity thinking fosters a humanized pedagogical practice that is concerned with how teaching is entangled with teachers' learning.

## Curriculum and systematization of thematic subject content blocks

There are questions that permeate the teacher's reflective process—What to teach? What should the students learn? How to select subject content? etc.—and are related to the curriculum. Souza et al. (25) reflect that the curriculum once had a concept that is now considered outdated, as a catalog of subjects that organizes knowledge in a logical sequence. As a counterpoint, before we think about what knowledge should be taught, we must think about whom it will be taught to, valuing the students, their life experiences and reality.

The curriculum can be defined, according to Sacristán (26), as what we know completely and can be taught or learned. The curriculum we wish to teach requires an organized selection of subject content that we seek for students to learn, and which regulate teaching practice in various methodological spheres. The curriculum converges with other educational demands, such as the organization of classes and the choice of methods. The curriculum is not static or immutable but it is flexible, dynamic, constructed historically and also in the pedagogical practice of teachers. It is through this perspective that the curriculum in school physical education has undergone theoretical-methodological transformations.

Sanches Neto (3) made an epistemological retreat when studying physical education curricular propositions and their complexity. The foundation comes from scientific understandings of the area and, at a deeper look, there are four converging dynamics between cultural elements, (inter)personal aspects of the body, movement and environmental demands. However, there are curricular documents supported by public policies—such as the BNCC (15) and the Document for Curricular Reference of Ceará (DCRC) (27)—linked to neoliberal interests and business sectors. In turn, these policies operate in the formulation of a more immediate minimum curriculum for the world of work, inducing the learning of skills of interest to these sectors (28).

Betti (9, 10) states that there is no doubt that the political purpose of the BNCC is to favor the interests of private entrepreneurs in the educational field, associated with quantitative evaluation so that schools are outsourced and it is viable to finance the pedagogical consultancy market by “commercializing” the education. The DCRC is linked to the BNCC, but has a regional characteristic, being a document that allows dialogue to complement the curriculum based on a broader concept of physical education, despite the valorization of cultural elements to the detriment of the demands of the body, movement and environment. In this sense, an autonomous group of teacher-researchers—understood as a community of knowledge—created a systematization of thematic subject content blocks to fill the gaps in physical education curricular proposals (5–7).

The systematization of thematic content blocks values convergence and interaction between knowledge that comes from the pedagogical practice of teachers themselves and different theoretical productions in the area, simultaneously, pointing to the physical education curriculum complexity (11). There is a critical political stance that supports systematization, analyzing social injustices and proposing knowledge focused on the complex demands that influence the historical process (29). The four thematic blocks converge on each other—cultural elements, movements, (inter)personal aspects and environmental demands—without hierarchies or overlapping of one dynamic over another, denoting complexity. As a coherence criterion for each class, it is suggested that the teacher teaches at least one topic from each block (6). We highlight complexity thinking because this proposal has non-linear aspects, that is, the blocks do not follow a standardized line. They configure their own times and spaces between the four dynamics, which are unique, inseparable and coexisting (3).

## Methodological choices

We used action research as a qualitative method through class observations, group meetings and field diaries, submitting them to thematic analysis (30). Quantitative data were added within the research descriptions, which involve, for example, the number of classroom observations and collaborative meetings. Furthermore, we used the methodological assumptions of Oliveira et al. (31) to create a collaborative network between participating teacher-researchers as they formed a research network based on the indications of each participant, taking as a starting point a school teacher entitled as teacher-researcher.

Action research is a consistent approach to address complex issues in physical education teaching such as democratic decision making towards social justice (32). Despite that — a decade ago — Rufino and Darido (33) found a low number of publications based on action research on physical education in schools, which also highlights the novelty of research in the area. Paradoxically, a justification for the lack of studies is that there is no tradition in research carried out by teachers who are not researchers of their own pedagogical practice.

For Venâncio et al. (34) it is noteworthy that nor all physical education related-research which is claimed as action research corresponds in fact to its methodological scope. However, as an attempt to demonstrate the categorization of areas that have used action research in school physical education, Rufino and Darido (33) point to the following themes: pedagogical intervention and teaching strategies (27% each), physical education teacher education (PETE) (17%), reflection on practice (13%), rupture with conservative perspectives and epistemology (7% each) and physical education in early childhood education (6%).

Even though physical education can advance more its usage of action research, it has proven to be an important strategy for expressing the opinion of social minorities and seeking a relationship of equity within the school. Some studies have specifically addressed teacher education, claiming that action research has promoted time and space for discussions with the potential for collaborative work given the intentionality of investigating practice (35). Then, there is an apparent consensus in understanding action research as an appropriate strategy for curricular systematization in physical education given the complexity involved in pedagogical innovation initiatives, considering contextual differences between schools settings (36–40).

Pedagogical action research—according to Venâncio et al. (34)—seeks an emancipatory and collaborative transformation between the teachers who participate in the research and the researcher in the light of ethical methods. Action research values temporality and evaluation in the continuity of the (self)formative processes of the participants involved. According to Franco (41, p. 491), there are methodological principles in action research, such as cyclical spirals, which involve “planning; action; reflection; search; resignification; replanning, actions increasingly adjusted to collective needs, reflections, and so on”.

We organized a collaborative network with physical education teachers who wanted to study their own pedagogical practice, assuming themselves as teacher-researchers. These teachers were previously chosen and, by crossing their recommendations from other teachers, we designed the collaborative network. The study was carried out with two physical education teachers from the public high school network in the municipality of Quixadá, one man and one woman.

The main researcher—first author Rener—observed four classes taught by each teacher-researcher and led two group meetings with them via *Google Meet*™ platform. Later, the research team planned new observations as a form of evaluation during a month and a final group meeting, also conducted by Rener, aimed at debating methodological processes. In summary, the research had four stages: (1) class observations, (2) collaborative meetings between teachers, (3) assessment observations, (4) final meeting to discuss all stages.

Data were recorded as field diary notes that — according to Vasconcellos and Francisco (42) — allow us to enter into realities that are not perceptible by other methodological instruments and which, in addition to the descriptive characteristic, must fulfill the reflective and analytical objective, providing the records after the proposed stages in the research and favoring the analysis through the subjective perception of the researcher. The data were analyzed and discussed collaboratively within a thematic analysis scope. According to Rosa and Mackedanz (30), themes can be identified through induction or deduction. We used deduction to describe overarching themes, highlighting in detail specific data from the analysis, such as class themes, convergence of knowledge and complexity through networks of interactions.

## Collaborative network design

The collaborative network was designed in a similar way to that proposed by Oliveira et al. (31). The researchers identified the concept of teacher-researcher in the literature and invited teachers who would fit this conceptual profile. Following the invitation, the chosen participants nominated other teachers with similar characteristics, thus forming a network of contacts. For ethical reasons, we guaranteed the anonymity of the teachers, identifying them by the letters A, D, H, K and R.

Initially, after approval by the ethics committee, the ground zero of the research was the first invitation addressed to teacher D, who already knew the first author, being entitled as teacher-researcher. This concept is based on Freire's (18) reflections on the teachers' need for permanent education to dialogue with questions about *praxis*, aiming for perception and assuming themselves as researchers. Based on this, teacher D was ground zero, that is, he received the first invitation to participate in the research and all other invitations started from that beginning.

Teacher D started his degree in 2007 and completed it in 2009. He has been a physical education teacher in the state public school system for over 15 years and has a Master's degree. He currently works at a school full-time but has worked at other schools in the city and neighboring municipalities. After accepting the invitation, teacher D indicated two possible contacts, teachers H and R, both recipients of a Specialist degree. Based on the recommendations, we contacted teacher H who started her degree in 2014 and completed it in 2018. She has been a physical education teacher since 2017 in a public high school and also agreed to participate in the research. Teacher R has accepted the invitation as well, however, due to his busy class schedules and the researcher's unavailability to accompany his professional activities thoroughly, it was not possible to reconcile the schedules.

Teacher R was asked to nominate other teachers, but he did not nominate anyone. We asked teacher H for nominations, who nominated teacher K and teacher A. Both are teachers at the same school. Teacher K did not agree to participate, justifying that she was hired for a few hours of physical education classes and these classes were at night in a district far from the headquarters in Quixadá. We contacted teacher A by phone and texting—via the *WhatsApp*™ application—but we did not receive any response. So, we completed our collaborative network design with teachers D and H, as there were no further indications. Then, we organized available times to carry out the research with the two teacher-researchers who decided to study their own pedagogical practice.

The research was previously approved by the Ethics and Research Committee of the Federal University of Rio Grande do Norte — as per code 6-038-255. The participating teachers confirmed their acceptance after signing the Free and Informed Consent Form (FICF) and also the authorization form for voice and video recording, documents that were previously approved. It is also important to highlight that before starting the research, we reflected on the number of participants and on the occasional fear of seeking more collaborators. However, this is a qualitative study and we understand that we must analyze the phenomena and subjectivities that surround them.

## Results and discussion

### Stage 1—class observations (four classes observed by each teacher)

The main objective of the observations was to analyze the complexity characteristics present in pedagogical practices and raise discussion topics, as well as the subject content explored and developed and its integration between the four thematic blocks. Table 1 presents a synthesis of the classes taught by teachers D and H.

**Table 1:** Classes synthesis.

Class 1—Teacher D—10 May 2023	Classes 2, 3 and 4—Teacher D—24 May 2023
*Central theme of the class*: Sport for people with disabilities (PwD);	*Central themes of the classes*: Accessibility;
*Secondary theme*: Goalball: characteristics and experiences;	*Secondary theme*: Society and PwD;
*Cultural elements*: Sport;	*Cultural elements*: Sport and Activities of Daily Living (AVD): quotes and examples;
*(Inter)personal aspects*: Human physiology;	*(Inter)personal aspects*: Human physiology: analysis of disabilities;
*Movements*: Basic motor skills and movement specialization;	*Movements*: Specificities of movements; types and degrees of disabilities;
*Environmental demands*: Physics and nature;	*Environmental demands*: Geography, politics and economy;
*Interaction networks*: Questions from students addressing other topics	*Interaction networks*: Analysis of themes and relationship with students’ reality
Class 1—Teacher H—15 May 2023	Classes 2, 3 e 4—Teacher H—24 May 2023
*Central theme of the class*: Organs and systems of the human body;	*Central themes of the classes*: Experiences of sport and games;
*Secondary theme*: Health;	*Secondary theme*: Not identified;
*Cultural elements*: AVD: relationship of the human body with physical exercise;	*Cultural elements*: Sport and game;
*(Inter)personal aspects*: Human anatomy: nomenclature of organs and internal structures, and health;	*(Inter)personal aspects*: Not identified;
*Movements*: Kinesiology: body movements and nomenclatures;	*Movements*: Not identified;
*Environmental demands*: Economy and politics: access to bodily practices and social inequality;	*Environmental demands*: Not identified;
*Interaction networks:* Relationship of the class theme with students’ life stories	*Interaction networks*: Interactions of student with student and of student with teacher

The objectives analyzed in the teachers' classes at this stage served to understand characteristics of complexity, with a dynamic look at the themes that the students wanted to explore, how the dynamics of knowledge converge with each other and how complex interactions are intertwined in the pedagogical practice.

### Stage 2—collaborative meetings between teachers (two collaborative meetings)

The systematization of thematic subject content blocks and the possibilities of linking them to the teachers' pedagogical interventions were discussed in group meetings. The objective through the action research is to bring the school closer while involving this theme, seeking a transformation of the teachers' reality (34) within the aspects that involve pedagogical practice and a critical emancipatory perspective. When presenting the systematization proposal, there was a direction for the next research stage, which is to know whether the teachers identified and understood the characteristics of complexity in their classes, whether there was a (self)critical reflection on their own pedagogical practice regarding DCRC, and whether the systematization of thematic subject content blocks has been included in their practices, even if intentionally proposed in the teaching plan or unintentionally.

Furthermore, topics such as the selection of subject content and dialogue with curricular documents were discussed. Teachers argued about the legislative guidelines mandatory to the educational sphere and their attempts to dialogue about those processes. The systematization of subject content blocks was presented and discussed about the various adversities of proposing themes in classes and planning. The pertinent dialogue between theoretical frameworks and pedagogical practices was directed during the collaborative meetings to suggest significant changes in the teachers' realities, valuing their education and life experiences. The meetings between teachers—in which the systematization of thematic subject content blocks was discussed—demonstrated participatory and collaborative aspects, such as the exchange of their ideas and experiences essential to promote changes in pedagogical practices.

### Stage 3—assessment observations (two evaluation observations)

At this stage we seek to understand how the systematization of thematic subject content blocks was included by teachers in their assessments. To do this, we observed their pedagogical practices in classes intended for assessment. Table 2 presents a synthesis of the assessments carried out by teachers D and H.

**Table 2:** Assessments synthesis.

Assessment 1—Teacher D—9 August 2023	Assessment 2—Teacher D—23 August 2023
*Central theme of the class*: Manifestations and dimensions of sport: education and participation;	*Central themes of the classes*: Manifestations and dimensions of sport, amateur and professional performance;
*Secondary theme*: Pre-sports games;	*Secondary themes*: Sport, media and society;
*Cultural elements*: Games and sports: experiences and explanation of concepts;	*Cultural elements*: Sport;
*(Inter)personal aspects:* Motor behavior: use of motor skills in experiences, not explained theoretically;	*(Inter)personal aspects*: Psychology, physiology, nutrition and health;
*Movements*: Combination and specialization of movements;	*Movements*: Capabilities and training: differences between amateur and professional;
*Environmental demands:* Politics and philosophy: laws and social norms that structure sport manifestations *and engagement;*	*Environmental demands*: Economy, management administration and politics;
*Interaction networks*: Active class participation	*Interaction networks*: Relationship between subject content and students’ reality
Assessments 1 and 2—Teacher H—21 August 2023	
*Central themes of the classes*: Muscular system and bodybuilding;	
*Secondary themes*: Health and fitness;	
*Cultural elements*: AVD: physical exercise bodily practices;	
*(Inter)personal aspects:* Anatomy, physiology and Kinesiology;	
*Movements*: Combination and specialization of movements;	
*Environmental demands*: Aesthetics, politics, sociology, among others;	
*Interaction networks*: Questions and objective participation	

This stage aimed to analyze how teachers mobilize knowledge—about the systematization of thematic subject content blocks as discussed in meetings—in their respective classes. Furthermore, we seek to understand whether there are convergences of themes in classes involving elements of culture, the body, movement and the environment, and whether they were pedagogically stimulated or not. By analyzing these perspectives, we try to understand how complexity is embedded and entangled in their classes. After the observations ended, we moved on to the last stage of research.

### Stage 4—final meeting to discuss the previous stages (one meeting)

The final group meeting had evaluative objectives and explanations of the results to the teachers, to understand whether and how the previous stages contributed and transformed their respective realities. Furthermore, we discussed how the subject content and pedagogical practice of teachers converged towards a complexity of knowledge involving physical education and the systematization of thematic subject content blocks, as well as the change in the participants' reality. In this last stage of research, a narrated statement was requested from the participating teachers about the changes that the research brought to their respective realities, as demonstrated in the methodological guidelines of action research (34) based on two questions: (1) What contributions did this research bring to your reality? (2) Did the systematization of thematic subject content blocks contribute to your pedagogical practice?

In summary, teacher D argued that, through participation, the planning of his classes began to take a different look at other themes that involve the systematization of thematic subject content blocks, a characteristic that, before, a theme from a specific block had a greater privilege of discussion, depending on the proposed class. Another contribution cited by teacher D was the need to study and delve deeper into new theoretical perspectives in the field of physical education and research. Teacher H agreed with the assignments and made similar reflections, pointing out a greater difference in pedagogical activities of planning classes, reflecting and valuing the knowledge that the students themselves bring to class. The need for (self)reflection in the teaching practice was also highlighted, being an enabler to encourage an assessment of their own pedagogical practices and seeking to understand the complexity that surrounds this field.

Through the principles of action research, this research included constant reflection on pedagogical practices throughout all stages, whether during class observation or discussions. The educative processes were considered and respected, seeking a reflective and complementary dialogue in each teacher's own pedagogical practice. The discussions about the classes took place with the participants themselves, seeking a transformation of their own reality. The results were interpreted and explained to the participants, especially in the last stage of the research. These changes that the research provides also affect the life of the teacher-researcher responsible for the action research. In this sense, the contributions from this research have affected beyond the participants.

It is essential to point out that there was a monitoring of classes and systematic records, according to the characteristics of each stage. In addition — as explained by the action research criteria by Venâncio et al. (34) — regarding moments of reflection and action, the research stages provided specific moments of reflection (stages 2 and 4) and action (stage 3). According to the theoretical basis of the methodology, there was also concern with the records of the teaching and learning processes. From this angle, narratives were captured (stage 4).

Through the conceptual contextualization of complexity and complexity thinking, the historical chronology of scientific and academic productions focused on school physical education and the analysis of the national and regional curriculum. It was noteworthy that the systematization of thematic subject content blocks has full conditions to assist and complement the teachers' pedagogical practice, dialoguing with the curriculum and valuing the theoretical advances of physical education in the educational context. According to Venâncio and Sanches Neto (24), the need to value physical education based on complexity is a qualitative shift against the intellectual simplism that permeates discourses about the area and this debate fosters collaborative dialogues between teacher-researchers.

However, these contributions and classes focused on complexity thinking require starting from the theoretical use of school planning possibilities, with pedagogical practices that intentionally aim to awaken themes and their convergences. By proposing planning in everyday practice, complexity has yet another stimulus to emerge through networks of interactions between teachers and students, making knowledge fluid through interaction, as it is at this moment that themes dialogue with the students' realities and their relationship to knowledge, valuing that their educational processes are important, creating interactive and collaborative dialogues (19).

When we approach the systematization of thematic subject content blocks (5, 6) and analyze how the themes are related and complement each other instead of designating the most important, we realize that there is the possibility of intervening in a complex way, understanding the issues brought up by the interactions within social spaces in a macro and micro sphere. From a theoretical and methodological point of view, the systematization of thematic subject content blocks proposes the use of at least one theme from each block per class. However, taking into account the minimum class time, sometimes the blocks will converge in a pedagogical sequence of classes, not necessarily in a specific class. Furthermore, within this reasoning from complexity thinking, sometimes in a class a theme from a specific block may stand out from the other blocks, but this does not mean that it is more important, but rather that a certain methodological approach requires a specific focus, without disregarding any other knowledge presented. On the other hand, this pathway requires attention, as highlighting a specific theme can distort other interactions.

One of the biggest difficulties about the thematic blocks highlighted by the participants was exploring themes involving environmental demands. Therefore, we conclude that if the lesson has critical, emancipatory, progressive perspectives and is focused on social justice, these themes emerge in the convergence of knowledge. In other words, it is necessary to expose the themes, provide experiences and dialogue—whenever possible, contextualizing them with the students' reality—analyzing the various environmental characteristics that involve these themes, as presented in the lesson discussions from this research. Sanches Neto et al. (6) provide reflection on the challenges of dealing with environmental demands in physical education classes, and one way to accomplish it is to make teaching more flexible and contextualize teaching linked to students' sociocultural issues, where teaching is aimed at social justice.

Betti (35) points to action research as a means of bringing the teachers' pedagogical practices closer to the realities of students. Therefore, teaching focused on environmental demands meets the need for investigations based on action research, while reflecting on real situations and acting towards them causes significant changes in pedagogical practice and leads to innovative concepts.

In relation to curricular documents, it was noticed that they direct the subject content towards the predominance of cultural elements, which can hinder the development of intersubjective knowledge related to the body, movement and environment. This can result in greater difficulty in developing the complexity of convergences and directing the elaboration of knowledge towards individual and subjective realities. Souza et al. (25) analyzed physical education teachers' pedagogical practices and noticed that the classes were mainly based on cultural elements and also on curricular guidelines. However, it is possible to rethink teaching themes from a complex perspective, expanding the horizons of knowledge and their convergences. For it is noteworthy that the curriculum is dynamic, flexible and historically constructed.

Nevertheless, curriculum documents linked to neoliberal interests operate in the formulation of a more immediate minimum curriculum for the world of work, with a fragmented vision of reality. The teacher-researchers' proposal to systematize thematic subject content blocks seeks to fill gaps both in physical education teacher education (PETE) and its curricular propositions. Therefore, dialoguing with such an open-ended systematization of thematic subject content blocks allows the manifestation of complex characteristics that are often made invisible by the documents that guide the teachers' pedagogical practices.

## Conclusion

We analyzed complex entanglements in the classes of two physical education teachers through the dynamics of cultural elements, (inter)personal aspects, movements and environmental demands, understanding and highlighting their contributions to pedagogical practice. Thus, we identified how the systematization of thematic subject content blocks can explain the limitations of curriculum documents that do not meet the complexity of classes.

In conclusion, it is understood that a critical view of the teacher-researcher on his/her own pedagogical practice can give new meaning to his/her teaching work, linking students with a view of valuing their knowledge and experiences, dialoguing and exploring these intervention possibilities. Furthermore, it is necessary to understand teaching as a complex activity, for the choices of themes, methodological strategies, ways of assessment, reflections and actions must be constantly revisited and reconstructed. In this sense, we believe that action research is a consistent tool for addressing the complexity of school physical education teaching and the complexity thinking entangled with it.

## Data Availability

The original contributions presented in the study are included in the article/supplementary material, further inquiries can be directed to the corresponding authors Rener Victor Oliveira de Souza, renervictor7@gmail.com.
